# Mechanical Stress Induce PG-E2 in Murine Synovial Fibroblasts Originating from the Temporomandibular Joint

**DOI:** 10.3390/cells10020298

**Published:** 2021-02-01

**Authors:** Ute Nazet, Laura Feulner, Dominique Muschter, Patrick Neubert, Valentin Schatz, Susanne Grässel, Jonathan Jantsch, Peter Proff, Agnes Schröder, Christian Kirschneck

**Affiliations:** 1 Department of Orthodontics, University Medical Centre of Regensburg, D-93053 Regensburg, Germany; laura.feulner@stud.uni-regensburg.de (L.F.); peter.proff@ukr.de (P.P.); agnes.schroeder@ukr.de (A.S.); christian.kirschneck@ukr.de (C.K.); 2 Centre for Medical Biotechnology, Department of Orthopaedic Surgery, Experimental Orthopaedics, University of Regensburg, D-93053 Regensburg, Germany; dominique.muschter@ukr.de (D.M.); susanne.graessel@ukr.de (S.G.); 3 Institute of Clinical Microbiology and Hygiene, University Hospital of Regensburg, D-93053 Regensburg, Germany; patrick.neubert@ukr.de (P.N.); valentin.schatz@ukr.de (V.S.); jonathan.jantsch@ukr.de (J.J.)

**Keywords:** temporomandibular joint, osteoarthritis, synovitis, inflammation, mechanical strain

## Abstract

Genetic predisposition, traumatic events, or excessive mechanical exposure provoke arthritic changes in the temporomandibular joint (TMJ). We analysed the impact of mechanical stress that might be involved in the development and progression of TMJ osteoarthritis (OA) on murine synovial fibroblasts (SFs) of temporomandibular origin. SFs were subjected to different protocols of mechanical stress, either to a high-frequency tensile strain for 4 h or to a tensile strain of varying magnitude for 48 h. The TMJ OA induction was evaluated based on the gene and protein secretion of inflammatory factors (*Icam-1*, *Cxcl-1*, *Cxcl-2*, *Il-1ß*, *Il-1ra*, *Il-6*, *Ptgs-2*, PG-E2), subchondral bone remodelling (*Rankl*, *Opg*), and extracellular matrix components (*Col1a2*, *Has-1*, collagen and hyaluronic acid deposition) using RT-qPCR, ELISA, and HPLC. A short high-frequency tensile strain had only minor effects on inflammatory factors and no effects on the subchondral bone remodelling induction or matrix constituent production. A prolonged tensile strain of moderate and advanced magnitude increased the expression of inflammatory factors. An advanced tensile strain enhanced the *Ptgs-2* and PG-E2 expression, while the expression of further inflammatory factors were decreased. The tensile strain protocols had no effects on the *RANKL/OPG* expression, while the advanced tensile strain significantly reduced the deposition of matrix constituent contents of collagen and hyaluronic acid. The data indicates that the application of prolonged advanced mechanical stress on SFs promote PG-E2 protein secretion, while the deposition of extracellular matrix components is decreased.

## 1. Introduction

The temporomandibular joint (TMJ) is the connection point of cranium and mandible and presents a unique articulated joint. Together with the articular disc, it forms a strong complex capable of carrying high loads and coping with immense pressure on the connected mandible [1]. Excessive mechanical exposure, genetic predisposition, or traumatic events provoke inflammation and arthritic changes, orofacial pain, articular noises, and limitations of mandibular movement [2,3].

Traumatic events can be caused by mechanical stimuli, comprising compressive or tensile, static or dynamic loading of varying frequency. Depending on the strain magnitude, frequency, and duration, an anabolic response will be triggered by intermediate magnitudes (characterised by cartilaginous matrix protein synthesis and accumulation), while extreme magnitudes of stress lead to a catabolic response in chondrocytes (MMPs expression, activity, and degradation products; for further details see review [4]). A common arthritic disease is osteoarthritis (OA), a multifactorially induced illness, characterized by changes in the chondrocyte as well as synoviocyte metabolism, regulating its emergence and progression. The synovial lining comprises two major cell populations, synovial macrophages and synovial-fibroblast-like cells. Together they form an important part of the synovial cavity, the synovial membrane. Characterised by dendritic processes, synovial fibroblasts (SFs) form a network of specialised cells in the matrix constituent production, maintaining the intimal joint interstitium and synovial fluid [5,6,7]. In addition to the articular cartilage subsistence, SFs play a mediating role in inflammation and OA progression. By secretion of proinflammatory cytokines and immune-receptors such as toll-like receptors (TLR), synovitis-induced chronic pain, articular cartilage degradation, and subchondral bone remodelling take place [8,9,10]. Apart from genetic predisposition and age, OA has a prevalence for women, previous traumatic events, repetitive high-impact loading, joint instability, and other systemic conditions [11,12,13,14]. Similarities in clinical signs and symptoms can be observed in OA and TMJ OA. Pain as well as joint function restriction is observed in patients suffering from TMJ OA [15]. While the cartilage status has an impact on TMJ OA emergence, the presence of juvenile idiopathic arthritis also has a prevalence [16,17]. For several years, interest in stromal-cell-like synovial fibroblasts has increased, as they have been identified to be key players in innate immune-system-response, inflammation-related processes, and intercellular actions, as well as principal performers involved in the OA development and progression [18,19,20]. A characteristic feature of inflammatory active synovial fibroblasts is the expression of proinflammatory cytokines such as IL-1β, IL-6, IL-8, and of enzymes with a matrix-degrading activity, the matrix metalloproteinases (MMPs). Elevated levels of these cytokines and MMPs promote further inflammatory and cartilage-degrading processes, as well as tissue vascularization [21,22,23,24]. Apart from cartilage degeneration, subchondral bone remodelling and increased bone metabolism takes place in orthodontically induced TMJ OA rat models [25], while a genetic model of TMJ OA showed increased levels of the receptor activator of nuclear factor kappa β ligand (RANKL) and osteoprotegerin (OPG) who are key players in osteoclast-activity regulating-processes [26]. By binding of RANKL to osteoclast precursor cells, a process of differentiation and maturation will be induced, resulting in mature osteoclasts being able to resorb the bone by acidification and via enzymes [27,28]. In contrast, OPG acts as a decoy receptor, expressing a higher binding affinity to RANKL and therefore, inhibiting osteoclast maturation [29]. By taking a look at the RANKL/OPG ratio, predictions on osteoclast differentiation can be given with a predominance of RANKL being an indicator of inflammatory processes [30,31]. During inflammation, changes of extracellular matrix composition and glycosaminoglycan deposition take place. The synthesis of collagen and hyaluronic acid is altered [32] and changes in the enzymatic activity of hyaluronic acid synthases, as well as a shift of type II to type I collagen can be observed [33,34,35].

In this study, we aimed to determine the influence of excessive mechanical stimuli on the metabolism of synovial fibroblasts originating from the temporomandibular joint and their impact on OA-related inflammatory processes.

## 2. Materials and Methods

### 2.1. Tissue Collection, Isolation, and Culture of Murine Synovial Fibroblasts

**Figure 1:**
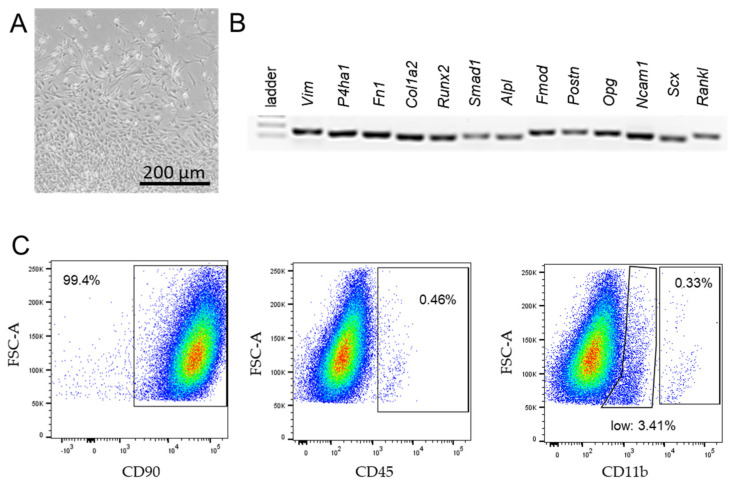
Characterisation of synovial fibroblasts from the temporomandibular joint of BL/6 mice. Spindle-shaped synovial fibroblasts growing out of the synovial tissue on collagen-I-coated flasks 2 weeks after extraction (**A**). The PCR expression analysis of fibroblast-specific-genes (**B**). Proportion of cells expressing cell-surface fibroblast-specific antigens (CD90.2) and leucocyte-specific antigens (CD45.2, CD11b) in the FACS analysis (**C**).

Wildtype BL/6N mice (female, 21 weeks old) were euthanised and prepared for the isolation of synovial fibroblasts from the temporomandibular joint. To this aim, the condylar head including the articular disc and attached synovial tissue were extracted under a microscope (Nikon SMZ 1500). For the isolation and culture of murine SF, the protocol given by Armaka et al. was applied with adaptions [36]. The tissue was harvested in ice-cold Dulbecco’s Modified Eagle’s Medium (DMEM, Sigma-Aldrich, Darmstadt, Germany, D5671), substituted with a 1% L-Glutamine solution (Sigma-Aldrich, Darmstadt, Germany, G7513) and 1% Antibiotic Antimycotic Solution (Sigma-Aldrich, Darmstadt, Germany, A5955). After harvesting, the tissue was washed two times with Dulbecco’s Phosphate-Buffered Saline (Gibco, Thermo Fisher Scientific, Waltham, MA, Germany, 14190-094), substituted with a 1% Antibiotic Antimycotic Solution (Sigma-Aldrich, Darmstadt, Germany, A5955). Then, the tissue was cut into small pieces and placed in DMEM, substituted with a 10% Fetal Bovine Serum (Pan Biotech, Aidenbach, Germany, P30-3302), 1% L-Glutamine solution (Sigma-Aldrich, Darmstadt, Germany, G7513), 1% Antibiotic Antimycotic solution, and digested with 0.1% Collagenase IV (Biochrom, Sigma-Aldrich, Darmstadt, Germany, C4-28) for 2 h at 37 °C. The samples were vortexed several times to improve tissue digestion. After digestion, the supernatant was removed by centrifugation and the supernatant as well as the remaining tissue pieces were transferred into separate collagen-I-coated (Enzo, Lörrach, Germany, ALX-522-435-0020) cell culture flasks with fresh cell culture medium. After 2 weeks, the outgrowth of synovial fibroblasts could be observed. Cells were characterised by their spindle-shaped appearance (Figure 1A), synovial-fibroblast-specific gene expression (*Vim, P4HA1*, *Fn1*, *Col1a2*, *Runx2*, *Smad1*, *Alpl*, *Fmod*, *Postn*, *Opg*, *Ncam11*, *Scx*, *Tnfsf11*; Figure 1B, Table 1) in combintaion with the fluorescence-activated cell sorting (FACS) analysis (Figure 1C). Only cells expressing > 90% CD90 (Biolegend, San Diego, CA, USA, 105327), <2.5% CD45 (Biolegend, San Diego, CA, USA, 109831), and <0.37% CD11b (eBioscience, Thermo Fisher Scientific, Waltham, MA, Germany, 45-0112-82) were used for further experiments [36,37]. Experiments were conducted with cells from passage 4 to maximum 7.

### 2.2. In Vitro Cell Culture Experiment Setup

**Figure 2:**
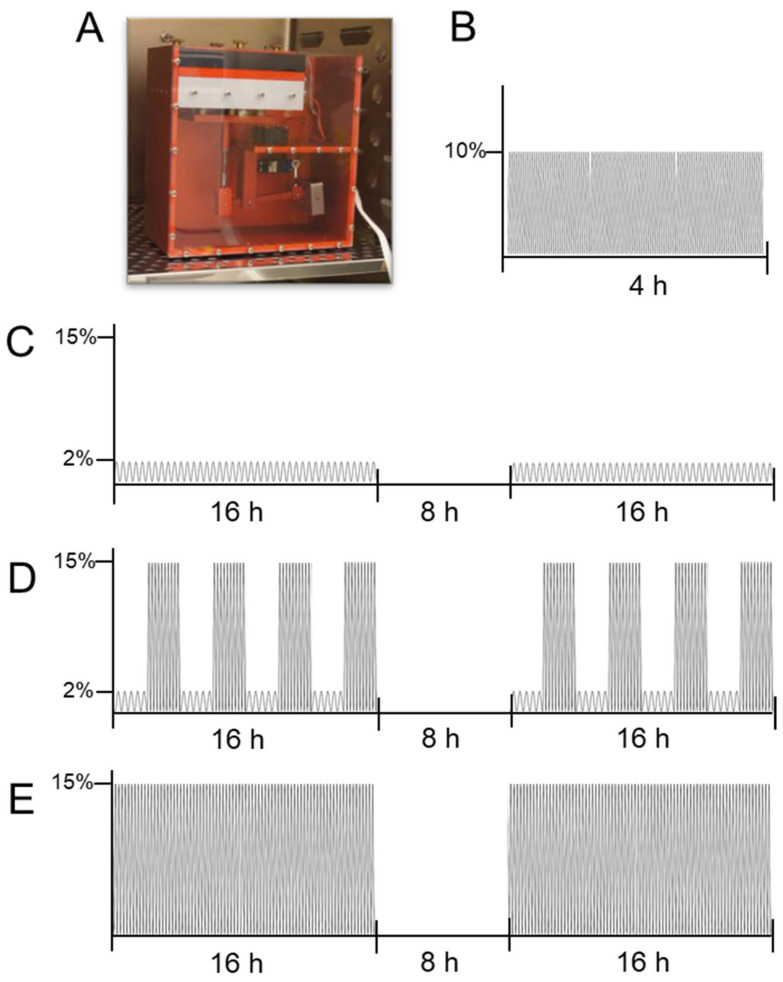
Visualisation of tensile loading protocols applied to synovial fibroblasts. Custom-made cyclic cell-stretching machine (**A**) consisting of a 6-well plate fitting slot and six stamps, which are simultaneously elongated and retraced according to a previously compiled script. The machine was used to expose cells to a short high-frequency tension (**B**) or prolonged cyclic tension (**C**: SM; **D**: SM/SA; **E**: SA).

Approximately 70,000 synovial fibroblasts per well were seeded onto a collagen-I-coated 6-well plate (BioFlex^®^, Dunn Labortechnik, Asbach, Germany, BF-3001C) and preincubated under cell culture conditions (37 °C, 5% CO_2_, water-saturated) for 24 h. Afterwards, a short and prolonged cyclic tension protocol was applied. To this aim, a custom-made cyclic cell stretching machine was used (Figure 2A).

The short high-frequency cyclic tension protocol was adapted from Muschter et al. [38] consisting of two groups: The control group, representing cells incubated under cell culture conditions for a total of 28 h, and the cyclic tension group, representing cells preincubated for 24 h followed by the cyclic tensile strain of 10% by a frequency of 1 Hz for a duration of 4 h (Figure 2B).

The prolonged cyclic tension protocol was adapted from Lohberger et al. [39] and consisted of four groups: The control group, which was incubated under cell culture conditions for a total of 72 h; the moderate stretching group (SM; Figure 2C), which was exposed to two blocks of low frequency (0.2 Hz), and low magnitude stretching (2% amplitude) for 16 h and a break of 8 h in-between; the intermediate group (SM/SA; Figure 2D), which was stretched 2 h with moderate stretching modalities and 2 h with advanced stretching modalities alternately until 16 h were completed, followed by an 8 h break and a repetition of alternate stretching and the advanced stretching group (SA; Figure 2E), which was exposed to two blocks of increased frequency (0.5 Hz) and high magnitude stretching (15% amplitude) for 16 h and a break of 8 h in-between.

### 2.3. RNA Isolation, cDNA Synthesis, Semiquantitative PCR, and Quantitative RT-PCR

RNA was isolated from cell pellets using the Trizol protocol (TriFast, peqGOLD, PEQLAB, Erlangen, Germany, 072319-30). The RNA pellet was reconstituted in nuclease-free water (Carl-Roth, Karlsruhe, Germany) and after photometrical adsorption measurements (280 and 260 nm; NanoDrop, Implen, Munich, Germany), the cDNA synthesis was performed as described before [40]. Briefly, 50 ng RNA were complemented with a 0.5 µL random hexamer primer (SO142; Life Technlogies, Thermo Fisher, Waltham, MA, USA), 0.5 µL oligo-dT18 primer (SO132; Life Technologies, Thermo Fisher Scientific, Waltham, MA, USA), 1xM-MLV-buffer (M531A; Promega, Madison, WI, USA), 0.5 µL dNTP mix (L785.1/2; Carl-Roth, Karlsruhe, Germany), 20 U RNase inhibitor (EO0381; Life Technologies, Thermo Fisher Scientific, Waltham, MA, USA), M-MLV reverse transcriptase (M1708; Promega, Madison, WI, USA), and nuclease-free water (T143; Carl-Roth, Karlsruhe, Germany) was added to a final volume of 10 µL. For reverse transcription, the samples were incubated 1 h at 37 °C followed by 2 min at 95 °C. For the semiquantitative PCR analysis, a mix of 2 ng cDNA template supplemented with 5 pmol specific oligonucleotides, 1xPCR Buffer + MgCl_2_ (14800100; Roche, Mannheim, Germany), 0.2 µL dNTPs (L785.1/2; Carl-Roth, Karlsruhe, Germany), and 0.5 U Taq-polymerase (39469200; Roche, Mannheim, Germany) was used. The final volume was set to 10 µL by the addition of nuclease-free water. Semiquantitative PCR was run with the following conditions: 5 min, 95 °C, 40 cycles 20 s 95 °C, 30 s 65 °C, and finally stored at 10 °C. The gene expression analysis was performed with the SYBR^®^ Green JumpStart™ Taq ReadyMix™ (S4438-500RXN; Sigma-Aldrich, Darmstadt, Germany) system and 1 ng cDNA template. The used oligonucleotides (Eurofins MWG Operon LLC, Huntsville, AL, USA; High Purity Salt Free Purification HPSF^®^) were designed according to the MIQE guidelines based on the gene sequences achieved from the Nucleotide database NCBI (Gene Bank, National Centre for Biotechnology Information; Table 1). The RT-qPCR reaction was performed in a Mastercycler^®^ ep realplex-S thermocycler (Eppendorf AG, Hamburg, Germany) with the following conditions: 95 °C for 5 min, 45 cycles of 95 °C for 10 s, 60 °C for 8 s, and 72 °C for 8 s, followed by a final melting curve analysis. The relative gene expression was analysed based on the C_q_ computation. Therefore, ∆C_q_ was calculated as the difference of C_q_ (target gene) subtracted by C_q_ (reference genes). As two reference genes were used for normalization, a combined C_q_ (geomean) was calculated as the square root of the sum of C_q_ (*Gapdh*) and C_q_ (*Ywhaz*) for the short high-frequency tensile strain protocol and C_q_ (*Hprt*) and C_q_ (*Sdha*) for the prolonged tensile strain protocol, which proved to be stably expressed under the experimental conditions tested (data not shown).

### 2.4. Protein and Glycosaminoglycan Analysis

#### 2.4.1. Enzyme-Linked Immunosorbent Assays (ELISA) 

For the protein expression analysis, the following kits were used according to the manufacturers’ instructions: Prostaglandin (MyBioSource, San Diego, CA, USA, Mouse Prostaglandin E2 ELISA, MBS266212), interleukin-6 (MyBioSource, San Diego, CA, USA, Murine IL-6 ELISA, MBS335514), osteoprotegerin (Thermo Fisher Scientific, Waltham, MA, USA, Mouse OPG (TNFRSF11B) ELISA Kit, EMTNFRSF11B), and Tnfsf11 (Thermo Fisher Scientific, Waltham, MA, USA, Mouse TRANCE (TNFSF11) ELISA Kit, EMTNFSF11).

#### 2.4.2. Total Collagen Content Assessment 

The total collagen amount was measured by means of the Total Collagen Assay Kit (BioVision, Milpitas, CA, USA, K218-100). After the supernatant sample hydrolysation and oxidation, the chromophore formation measured at an absorbance at 560 nm directly indicated the collagen quantity.

#### 2.4.3. Liquid-Chromatography Glycosaminoglycan Analysis

The hyaluronic acid content was evaluated via the high-performance liquid chromatography (HPLC) separation (column: Sphere-Image 80-5 SAX, Knauer, Berlin, Germany; equipment: Shimazdu). The unsaturated disaccharides generated from hyaluronic acid were analysed after an enzymatic treatment. Therefore, glycosaminoglycan precipitation was performed with the supernatant. After freeze-drying, the pellets were reconstituted in 300 µL H_2_O_dd_, supplemented with 900 µL EtOH and stored over night at −20 °C. After centrifugation (5000 rpm, 5 min, 4 °C), the pellets were harvested, resuspended in 300 µL 0.1 M ammoniumacetate containing 3 µL 109 U/mL proteinase K (Sigma-Aldrich, Darmstadt, Germany, P4850-50ML), and incubated for 2 h at 55 °C on a rotary shaker at 700 rpm. Finally, the samples were heated for 5 min at 100 °C for proteinase inactivation. Then, 900 µL EtOH was added, samples were inverted and stored over night at −20 °C. Pellets were harvested via centrifugation (5000 rpm, 5 min, 4 °C) and resuspended in 50 µL H_2_O_dd_. For the HPLC analysis, 10 µL samples were supplemented with 18 µL buffer (0.1 M Tris; 0.15 M NaAcetate, pH 8.0) and 2 µL chondroitinase ABC (Sigma-Aldrich, Darmstadt, Germany, C3667), and digested over night at 37 °C. HPLC detection was performed at 232 nm and separation was detectable within 0 to 15 min with 10 mM NaH_2_PO_4_ (pH 4.0) and within 15 to 35 min with 10 mM NaH_2_PO_4_ (pH 4.0) to 33% 750 mM NaH_2_PO_4_ (pH 4.0) with a flow rate of 1.2 mL/min.

### 2.5. Statistical Analysis

The program GraphPad Prism 8 (GraphPad Software, San Diego, CA, USA) was used for statistical analysis. All tests were performed as two-sided and significance was set at *p* ≤ 0.05. The normal distribution of data was determined using Shapiro-Wilk tests. Depending on the data distribution, either an unpaired t-test or a Welch-corrected t-test was conducted for a comparison of the two groups. Comparing more than two groups, either a Welch-corrected ANOVA (analysis of variance), followed by Games-Howell multiple comparison tests or an ordinary ANOVA followed by Holm Sidak’s multiple comparison tests, was performed. For data transparency, single datapoints were displayed in figures with error bars representing the mean and standard error of the mean.

## 3. Results

### 3.1. Effects of Cyclic Tensile Strain on the Expression of Inflammatory Mediators in Synovial Fibroblasts of the Temporomandibular Joint

Synovial fibroblasts of the temporomandibular joint are expected to be exposed to a frequent tensile strain on a daily basis. Progressing arthritic changes are indicated by increased levels of local and systemic inflammation.

**Figure 3:**
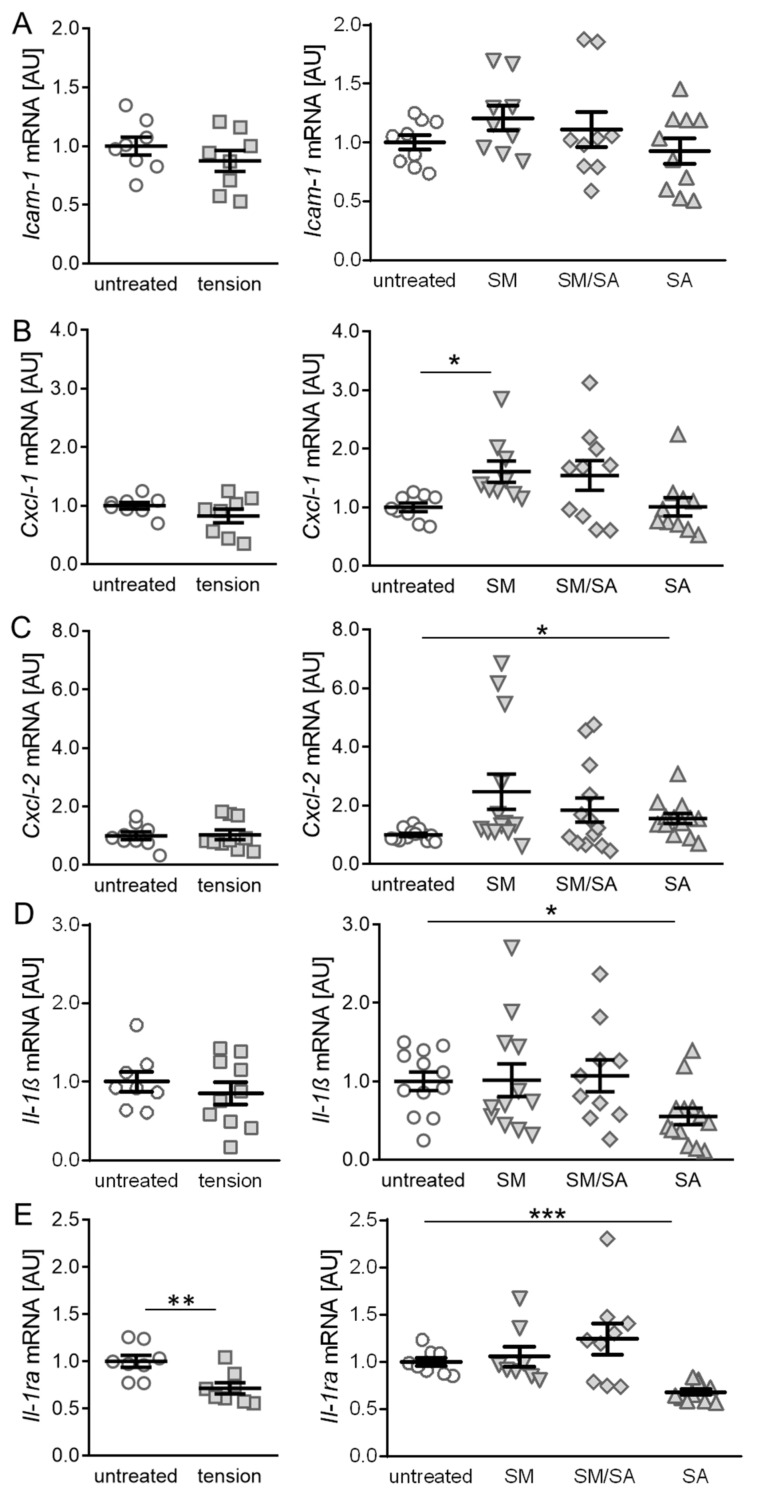
Effects of different tensile loading protocols on genes involved in the immune cell recruitment. The relative gene expression of *Icam-1* (**A**), *Cxcl-1* (**B**), *Cxcl-2* (**C**), *Il-1ß* (**D**), and *Il-1ra* (**E**). AU: Arbitrary units; SM: Moderate; SM/SA: Intermediate; SA: Advanced. *n* ≥ 5, * *p* ≤ 0.05, ** *p* ≤ 0.01, *** *p* ≤ 0.001.

We first focused on genes involved in the immune cell recruitment and analysed the effects of various tensile protocols. The *Icam-1* gene encodes for a cell surface glycoprotein, which is induced by cytokines and involved in leukocyte recruitment [41,42]. The gene expression of *Icam-1* was neither affected by the short high-frequency tensile strain (*p* = 0.3031; Figure 3A) nor the prolonged tensile strain (SM: *p* = 0.3702; SM/SA: *p* = 0.9050; SA: *p* = 0.9316). Next, we investigated the gene expression of *Cxcl-1* and *Cxcl-2*, which encode chemo-attractants for neutrophils and leukocytes. The gene expression of *Cxcl-1* or *Cxcl-2* was not affected by the short tensile strain protocol (*Cxcl-1*: *p* = 0.2024, *Cxcl-2*: *p* = 0.8681; Figure 3B,C). The prolonged moderate tensile strain (SM) increased the *Cxcl-1* expression (*p* = 0.0432), while the intermediate (SM/SA: *p* = 0.2253) or advanced (SA: *p* > 0.9999) stretching had no impact on the *Cxcl-1* gene expression (Figure 3B). In contrast, we detected no effects of moderate (SM: *p* = 0.1244) or intermediate (SM/SA: *p* = 0.2309) tensile loading, while advanced stretching (SA: *p* = 0.0315) increased the *Cxcl-2* expression significantly (Figure 3C).

Next, we analysed the gene expression of the pro-inflammatory cytokine interleukin 1β (*Il-1β*). No effects on the *Il-1β* gene expression were present, when treating synovial fibroblasts with the short high-frequency tensile strain (*p* = 0.4471; Figure 3D). However, similar to the prolonged tensile strain protocol, the gene expression of *Il-1β* was decreased, when exposing cells to advanced stretching (SA: *p* = 0.0448) with no effects of moderate (SM: *p* = 0.9999) or intermediate (SM/SA: *p* = 0.9903) tensile strain (Figure 3D). The IL-1β-mediated inflammation is induced by specific binding to the IL-1 receptor. When anti-inflammatory processes are activated, the IL-1RA expression is increased in order to outcompete IL-1β by binding to the IL-1 receptor. Therefore, the expression of *Il-1ra* was analysed and surprisingly revealed a significant decrease in the *Il-1ra* gene expression (*p* < 0.0056) when synovial fibroblasts were treated with the short high-frequency tensile strain (Figure 3E). The gene expression of *Il-1ra* was reduced with the advanced tensile strain (SA: *p* < 0.0001), while moderate (SM: *p* = 0.9655) or intermediate (SM/SA: *p* = 0.5167) loading protocols failed to impact on the *Il-1ra* gene expression (Figure 3E).

**Figure 4:**
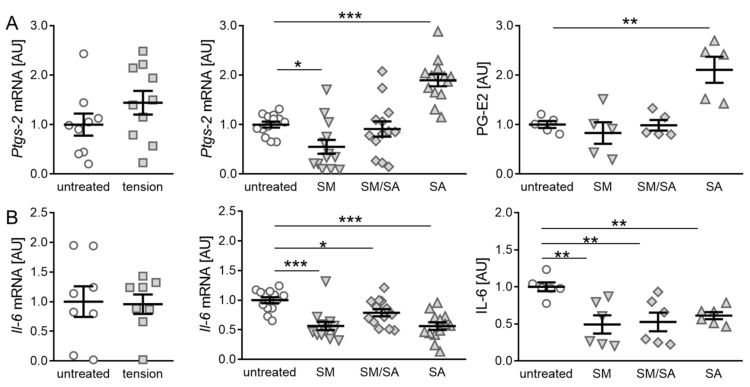
Synovial-fibroblast-mediated expression of inflammatory markers induced by various tensile loading protocols. Expression profiles of the inflammation indicator *Ptgs-2*, PG-E2 (**A**), and the inflammatory cytokine IL-6 (**B**) induced by the short high-frequency tensile strain or prolonged tensile strain, respectively. AU: Arbitrary units; SM: Moderate; SM/SA: Intermediate; SA: Advanced. *n* ≥ 5, * *p* ≤ 0.05, ** *p* ≤ 0.01, *** *p* ≤ 0.001.

The gene expression of *prostaglandin synthase-2* (*Ptgs-2*) was not affected by the short high-frequency tensile strain (*p* = 0.1982; Figure 4A), while fibroblasts exposed to the prolonged moderate tensile strain reduced the *Ptgs-2* gene expression (SM: *p* = 0.0410; Figure 4A). In contrast, the advanced tensile strain increased the *Ptgs-2* gene expression (SA: *p* ≤ 0.0001) combined with a significantly increased prostaglandin E2 (PG-E2) synthesis (*p* = 0.0170). However, no effect on the PG-E2 production was observed by the moderate and intermediate tensile strain (SM: *p* = 0.7648; SM/SA: *p* = 0.9514; Figure 4A). Next, the effect of the tensile strain on the inflammatory cytokine interleukin-6 (IL-6) was analysed. As for the inflammation mediator *Ptgs-2*, no effects on the *Il-6* gene expression were observed, when exposing cells to the short high-frequency tensile strain (*p* = 0.8946; Figure 4B). In contrast, a reduced *Il-6* gene expression was detected, when fibroblasts were exposed to the prolonged moderate (SM: *p* = 0.0001), intermediate (SM/SA: *p* = 0.0355), and advanced tensile strain (SA: *p* < 0.0001). Accordingly, this was accompanied by a significantly reduced IL-6 protein secretion (SM: *p* = 0.0039; SM/SA: *p* = 0.0046; SA: *p* = 0.0096; Figure 4B).

### 3.2. Effect of Cyclic Tensile Strain on RANKL/OPG-Mediated Osteoclast Activation

**Figure 5:**
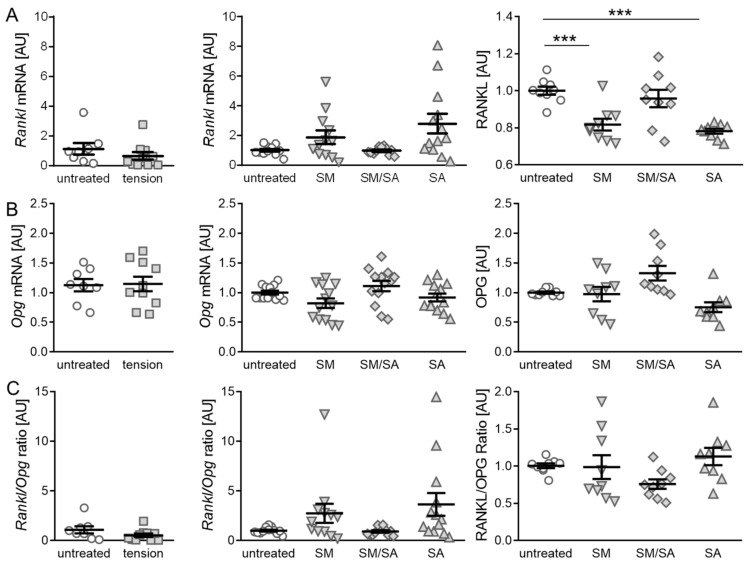
Synovial-fibroblast mediated expression of RANKL and OPG induced by various tensile loading protocols. The gene and protein expression of RANKL (**A**) and the corresponding decoy receptor OPG (**B**), as well as the RANKL/OPG ratio (**C**) after the short high-frequency tensile strain or prolonged tensile strain. AU: Arbitrary units; SM: Moderate; SM/SA: Intermediate; SA: Advanced. *n* ≥ 6; *** *p* ≤ 0.001.

The initiation of subchondral bone remodeling is a further key factor in the induction and progression of osteoarthritic changes. An analysis of RANKL and OPG, which acts as a RANKL decoy receptor, can give a hint on the bone remodelling activity. Therefore, an analysis of RANKL as well as the OPG expression was performed. The gene expression of *Rankl* showed no significant differences, when cells were exposed to the short high-frequency cyclic tension (*p* = 0.3239; Figure 5A). Moreover, when treated with the prolonged cyclic tension, no differences in gene expression were determined (SM: *p* = 0.2850; SM/SA: *p* = 0.9911; SA: *p* = 0.0808). In contrast, the analysis of RANKL protein secretion revealed a significant downregulation, when treated with the moderate (SM, *p* = 0.0004) or advanced tension (SA, *p* ≤ 0.0001), while the intermediate tension (SM/SA, *p* = 0.3415) caused no effects. The gene expression of *Opg* was not affected by the short high-frequency tension (*p* = 0.9031; Figure 5B). The prolonged protocol also had no significant effect on the *Opg* gene expression (SM: *p* = 0.2115; SM/SA: *p* = 0.4648; SA: *p* = 0.4648). Accordingly, we detected no changes in the OPG protein secretion with the investigated loading protocols (SM: *p* = 0.9965; SM/SA: *p* = 0.1096; SA: *p* = 0.0722; Figure 5B). As osteoclast differentiation is dependent on the interaction of RANKL and its decoy receptor OPG, a ratio between RANKL and OPG was determined. As expected by the expression of the single attributes, the short high-frequency tensile strain had no significant effects (*p* = 0.1728; Figure 5C). Moreover, the ratio of *Rankl* and *Opg* gene expression calculated for the prolonged tension protocol showed no significant changes (SM: *p* = 0.3209; SM/SA: *p* = 0.9572; SA: *p* = 0.1519). Accordingly, the ratio of RANKL and OPG protein secretion was not affected by the prolonged stretching protocols (SM: *p* = 0.9074; SM/SA: *p* = 0.2944; SA: *p* = 0.6469).

### 3.3. Diminished Matrix Constituent Deposition by the Prolonged Cyclic Tensile Strain

**Figure 6:**
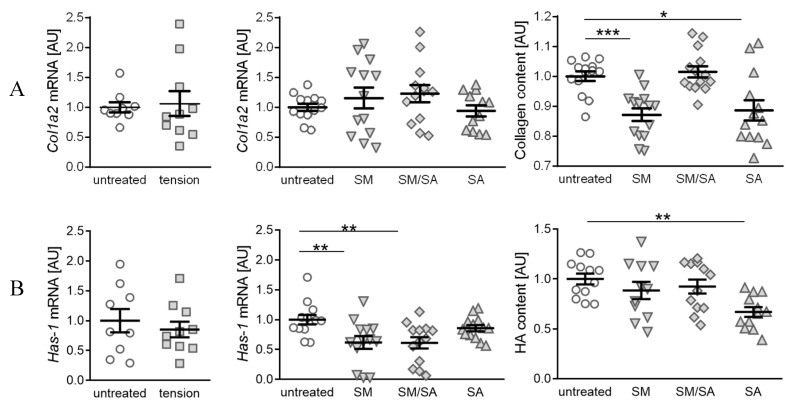
Synovial-fibroblast-mediated expression of matrix constituent products induced by various tensile loading protocols. Analysis of the gene expression profile of *Col1a2* and soluble collagen components (**A**), and the gene expression of *Has-1* and soluble glycosaminoglycan hyaluronan acid deposition (**B**) induced by the short high-frequency tensile strain or prolonged tensile strain, respectively. AU: Arbitrary units; SM: Moderate; SM/SA: Intermediate; SA: Advanced. *n* ≥ 6, * *p* ≤ 0.05, ** *p* ≤ 0.001, *** *p* ≤ 0.0001.

Synovial fibroblasts regulate the matrix production and joint lubrication homeostasis. While healthy joints are characterised by collagen II presence, it can be observed that the advanced arthrosis is associated with increasing collagen I levels [33,35]. An analysis of collagen-1-alpha-2 (*Col1a2*) gene expression revealed no differences in the gene expression, when treated with the short high-frequency tension (*p* = 0.7989) or different prolonged tension protocols (SM: *p* = 0.6323; SM/SA: *p* = 0.4952; SA: *p* = 0.7247; Figure 6A). However, the analysis of the conditioned medium for soluble total collagen deposition revealed significantly diminished collagen fragment contents in samples treated with either the moderate (SM: *p* = 0.0003) or advanced tension (SA: *p* = 0.0310), while intermediate stretching had no effect (SM/SA: *p* = 0.9213). Hyaluronic acid (HA) is an important constituent of joint lubrication and is produced by synovial fibroblasts [24]. While three different hyaluronic acid synthases (HAS1-3) are known, HAS-1 generally expresses a low level of enzymatic activity, which is impaired with arthritic changes [34]. The influence of cyclic tension was tested on the *Has-1* gene expression as well as on hyaluronic acid fragments. We observed no differences in the *Has-1* gene expression, when treating synovial fibroblasts with the short high-frequency tension (*p* = 0.5293; Figure 6B). In contrast, the prolonged tension protocol led to decreased *Has-1* gene expression levels. The moderate (SM: *p* = 0.0070) as well as intermediate tensile strain (SM/SA: *p* = 0.0070) significantly diminished the *Has-1* gene expression, while the advanced tensile strain had no effect (SA: *p* = 0.2443). The analysis of the soluble HA fragment deposition in the conditioned medium, however, revealed a significantly decreased presence of the glycosaminoglycan HA after the advanced tensile strain (SA: *p* = 0.0024), while the moderate (SM: *p* = 0.3956) and mixed tensile strain (SA: *p* = 0.4087) had no effects.

## 4. Discussion

Findings of a population-based study with the aim to identify the prevalence and incidence of clinical diseases observed signs of MRI-diagnosed OA in the TMJ in nearly 25% of the participants [43,44]. While TMJ OA is painful in the early stage, OA progression as well as an increasing age lead to a decrease of clinical signs and pain, whereas radiological imaging reveals disc destruction and OA progression [45]. The TMJ is one of the most used joints due to mastication; thus exceeding mechanical stress is a risk factor of OA. In healthy TMJ joints, the disc acts as a stress absorber, while enabling cooperative movement with condylar repositioning during jaw movement. When exceeding stress affects the joint, disc displacement may occur with transmission to the surrounding tissues [46]. As the TMJ is—apart from the knee and clavicular joints—the only synovial joint with an articular disc, research in this topic reveals important insights in understanding osteoarthritis [46]. TMJ OA is an important disease in orthodontics and a fundamental factor in the reduction of quality of life. In patients suffering from temporomandibular disorders, an altered expression of the cytokines IL-1β and IL-6 and the endogenous negative-feedback regulator IL-1RA was found. While blood plasma of patients with temporomandibular disorders (TMD) showed elevated levels of IL-1RA and IL-8 [47], synovial fluid samples of TMD patients showed increased levels of IL-1β, IL-2, IL-6, IL-8, as well as the IL-6 soluble receptor, while IL-1RA was not induced [48,49]. Furthermore, the synovium undergoes degenerative changes, while maintaining inflammatory processes during TMJ degeneration [50]. A short high-frequency tensile strain model was applied in order to analyse the effects caused by single events of high frequency stretching. Tensile-strain-treated cells were harvested and processed directly after the stress application. Our data demonstrate that the short duration of high-frequency loading had no effect on the pro-inflammatory gene expression or extracellular matrix composition. In order to achieve a more naturally occurring stress, the prolonged cyclic tension protocol was used. The effect of long-time occurring mechanical stress was investigated using various tensile strain amplitudes and frequencies. No explicit effects on the inflammation and matrix remodelling occurred, when applying low loading conditions by the moderate tensile stress protocol, except for diminished levels of IL-6 and collagen deposition. With the increasing frequency and amplitude, pain- and swelling-related processes might be activated, indicated by the elevated *Ptgs-2* gene and PG-E2 protein levels. Furthermore, the activated *Ptgs-2*/PG-E2 pathway exerts cartilage turnover favouring processes, by inducing the RANKL transporter on the chondrocyte cell membrane [51] and downregulating the matrix components gene expression, while inhibiting the proteoglycan production [52]. In their study, the TMJ cartilage tissue exposed to mechanical stress also induced the *Ptgs-2* expression, while the IL-1β expression was not altered [53]. In our case, advanced stretching for 48 h reduced inflammatory processes, detectable by significantly decreased levels of IL-6 and *Il-1β*. By increasing the IL-1RA plasma levels, an accommodation in TMJ occurs in order to reduce the activation of related stress pathways, as well as inflammation and pain [47,54]. In general, IL-1RA competes with IL-1α and IL-1β in binding the IL-1 receptor. By an 100-fold increased IL-1RA expression, a 50% inhibition of IL-1 induced response can be achieved [55]. Furthermore, the increasing IL-1RA levels impede mechanically induced hyperalgesia [56] and indicate the development of temporomandibular disorders [47], while increasing CXCL-2 levels attenuate in osteoblast differentiation processes [57] and can indicate early inflammation [58]. In our experimental setup, the *Il-1ra* gene levels were decreased. In combination with the decreased levels of *Il-1β*, the applied mechanical strain was not able to induce the Il-1 mediated inflammation. Assuming that, in order to prevent an inflammatory breakdown of the cartilage and immune infiltration, synovial fibroblasts express anti-inflammatory pathways triggered by ongoing mechanical stimulation, while failure will promote TMJ OA [59]. Interestingly, the alternating combination of the moderate and advanced strain (SM/SA) had no effects on cytokine expression. This suggests that a long incubation exceeding 2 h of advanced stretching is required for TMJ synovial fibroblasts to alter cytokine-related pathways. It may be possible that the constant monotone mechanical strain of advanced amplitude is required to sustain cytokine depletion. While under usage (SM) of the TMJ seemed to support angiogenesis and wound healing processes by increased *Cxcl-1* levels [60], the TMJ seemed to benefit from mechanical loaded synovial fibroblasts by the depleted proinflammatory cytokine expression.

An experimental application of a mechanical load on osteoblasts was shown to be associated with decreased OPG levels and OPG/RANKL ratio, an induction of chondrocyte apoptosis and with matrix degradation mediated by matrix metalloproteinases [61]. Similar to mechanically loaded condylar cartilage expressing diminished RANKL levels [62], TMJ synovial fibroblasts showed a significant diminished RANKL expression, when treated with the prolonged advanced tensile strain. While the OPG expression was not altered, the ratio of RANKL/OPG showed no differences to untreated cells. Moreover, the moderate cyclic tensile strain induced no effects, similar to the RANKL/OPG ratio showed by the alternating cyclic tensile strain no induction of subchondral bone remodelling mediated via osteoclast activation was indicated. In TMJ OA, the RANKL expression correlates with the proinflammatory cytokine expression, while inducing osteoclastogenesis and bone resorption processes [63]. The diminished RANKL expression fits the observed depletion in the proinflammatory cytokine expression and indicates protection of bone resorption processes. 

Synovial fibroblasts play an important role in the lubrication of articular cartilage surfaces. Hyaluronan acid, a major lubricant macromolecule, thereby provides viscosity as well as outflow buffering [64,65]. During injuries and arthritic changes, decreasing levels of HA can be observed [66,67,68], while the synthesis and secretion is altered [32]. With increasing levels of TGF-β and IL-1β, synovial fibroblasts experience a dose-dependent upregulation of HAS1 in order to regulate acute inflammation following injury [69,70,71], while under normal conditions, HAS1 experiences the lowest enzymatic activity compared to HAS2 and HAS3 [72]. We expected a mechanically induced inflammation, especially when compiling the SA protocol. However, the applied protocols revealed a tension-mediated downregulation of *Has-1* gene expression indicating an anti-inflammatory environment. The deposition of hyaluronic acid can be impaired by the enzymatic activity of hyaluronidases. As the *Has-1* expression does not differ from control cells and intact hyaluronic acid is part of the insoluble extracellular matrix of synovial fibroblasts, we assume that either the reduced displacement of insoluble hyaluronic acid occurs in advanced stretching, or hyaluronidases show an increased activity in the advanced stretching condition. Apart from the hyaluronic acid, collagen is a major component of the extracellular matrix, synthesized by synovial fibroblasts. When inflammatory cells infiltrate tissues, an altered collagen synthesis and deposition co-localized with hyaluronic acid deposition can be observed [73,74,75]. In the case of arthritic changes, increasing levels of collagen type I and collagen type II can be detected [33], while high molecular weight collagen peptides induce the collagen type I mRNA expression in fibroblasts [76]. Mechanical stimulation, however, did not alter the collagen type I mRNA expression, while the soluble total collagen content was diminished in the supernatant of cells treated with prolonged moderate and advanced stretching protocols. In accordance with the assumption, that collagen accumulation may be impaired with the hyaluronic acid present, the reduced collagen deposition may be caused by the diminished hyaluronic acid fragment presence in the supernatant of advanced stretched synovial fibroblasts. It is also possible, that moderate and advanced stretching reduce the displacement of insoluble collagen. However, the unaltered collagen type I mRNA expression and collagen deposition do not indicate an inflammatory status in TMJ synovial fibroblasts exposed to the mechanical strain. Furthermore, by decreasing the levels of deposited collagen and hyaluronic acid, the viscosity of the synovial fluid will be diminished and therefore, provide better nourishing attributes for the articular cartilage, while losing shock-absorbing attributes during prolonged advanced mechanical stress. Due to the disc position in the joint capsule, two compartments are formed (superior joint cavity and inferior joint cavity). During the masticatory movement, the disc moves inside the joint capsule restricted by ligaments. This movement induces a synovial fluid pump in the TMJ, leading to the circulation of synovial fluid inside the joint capsule [77,78]. As the synovial fluid is produced by synovial fibroblasts, the reduction of deposited hyaluronic acid and collagen fragments may result by the advanced stretching, in order to improve synovial fluid movement attributes, while being pumped inside the joint cavities. 

## 5. Conclusions

The applied cyclic mechanical tensile strain protocols did not cause a clear and distinct arthritic phenotype in synovial fibroblasts originating from the murine temporomandibular joint. However, the advanced mechanical strain induced PG-E2 protein expression, while the deposition of extracellular matrix components and RANKL mediated osteoclastogenesis was reduced.

## Figures and Tables

**Table 1:** Primer sequences for reference and target genes used in RT-qPCR and semiquantitative PCR.

Gene-Symbol	Gene Name	5′-Forward Primer-3′	5′-Reverse Primer-3′
*Alpl*	alkaline phosphatase	GGGGTACAAGGCTAGATGGC	AGTTCAGTGCGGTTCCAGAC
*Col1a2*	collagen, type I, alpha 2	TGGCCCCAATGGATTTGCTG	CCTTAGGCCCTTTGGTTCCC
*Cxcl-1*	C-X-C motif chemokine ligand 1	CTGGGATTCACCTCAAGAACATC	CAGGGTCAAGGCAAGCCTC
*Cxcl-2*	C-X-C motif chemokine ligand 2	TTAAAAACCTGGATCGGAACCAA	GCATTAGCTTCAGATTTACGGGT
*Fmod*	fibromodulin	CCTCCTGTCAACACCAACCTGG	TTCCCATCCAGGCGTAGCAC
*Fn-1*	fibronectin 1	AGCCAGGAACCGAGTACACC	AGCCAGGAACCGAGTACACC
*Gapdh*	glyceraldehyde-3-phosphate dehydrogenase	GTCATCCCAGAGCTGAACGG	ATGCCTGCTTCACCACCTTC
*Has-1*	hyaluronan synthase 1	TGACAGGCACCTCACCAACC	TGGCTCAACCAACGAAGGAAGG
*Hprt*	hypoxanthine guanine phosphoribosyl transferase	AGCTTGCTGGTGAAAAGGAC	AGTCAAGGGCATATCCAACAAC
*Icam-1*	intercellular adhesion molecule 1	GTGATGCTCAGGTATCCATCCA	CACAGTTCTCAAAGCACAGCG
*Il-1β*	Interleukin-1 β	GTGTAATGAAAGACGGCACACC	ACCAGTTGGGGAACTCTGC
*Il1-ra*	Interleukin-1 receptor antagonist	GCTCATTGCTGGGTACTTACAA	CCAGACTTGGCACAAGACAGG
*Il-6*	Interleukin-6	ACAAAGCCAGAGTCCTTCAGAG	GAGCATTGGAAATTGGGGTAGG
*Ncam-1*	neural cell adhesion molecule 1	GTCACTCTGACCTGTGAAGCC	CACCATGTGCCCATCCAGAG
*Opg*	osteoprotegerin	CCTTGCCCTGACCACTCTTAT	CACACACTCGGTTGTGGGT
*P4ha-1*	prolyl 4-hydroxylase subunit alpha 1	GTCTGGCTACGAAGACCCTGTG	GGGGCTCATACTGTCCTCCAAC
*Postn*	periostin	TCATTGAAGGTGGCGATGGTC	AACGGCCTTCTCTTGATCGTC
*Ptgs-2*	prostaglandin-endoperoxide synthase 2	TCCCTGAAGCCGTACACATC	TCCCCAAAGATAGCATCTGGAC
*Rankl*	tumor necrosis factor superfamily, member 11	AAACGCAGATTTGCAGGACTC	CCCCACAATGTGTTGCAGTTC
*Runx-2*	runt related transcription factor 2	GACGTGCCCAGGCGTATTTC	CACCTGCCTGGCTCTTCTTAC
*Scx*	scleraxis	AGAACACCCAGCCCAAACAG	ATCGCCGTCTTTCTGTCACG
*Sdha*	succinate dehydrogenase complex, subunit A, flavoprotein	AACACTGGAGGAAGCACACC	AGTAGGAGCGGATAGCAGGAG
*Smad-1*	SMAD family member 1	CGGGTTCGAGACCGTGTATG	GGGGTGCTGGTAACATCCTG
*Vim*	vimentin	TTCTCTGGCACGTCTTGACC	GCTTGGAAACGTCCACATCG
*Ywhaz*	tyrosine 3-monooxygenase/tryptophan 5-monooxygenase activation protein	AATGCTTCGCAACCAGAAAGC	TGGTATGCTTGCTGTGACTGG

## Data Availability

All data is available upon request from the corresponding author.
